# BIRC5 as a prognostic and diagnostic biomarker in pan-cancer: an integrated analysis of expression, immune subtypes, and functional networks

**DOI:** 10.3389/fgene.2024.1509342

**Published:** 2024-12-05

**Authors:** Guoyu Li, Yanghao Wang, Weizhou Wang, Guodong Lv, Xiang Li, Jingying Wang, Xiuyu Liu, Daolang Yuan, Shoujun Deng, Dingyun You

**Affiliations:** ^1^ Department of Colorectal Surgery, Yunnan Cancer Hospital, Kunming, Yunnan, China; ^2^ Department of Pathology, The First Affiliated Hospital of Kunming Medical University, Kunming, Yunnan, China; ^3^ Department of Orthopedics, The First Affiliated Hospital of Kunming Medical University, Kunming, Yunnan, China; ^4^ Clinical College, Kunming Medical University, Kunming, Yunnan, China; ^5^ Department of Thoracic Surgery, Yanan Hospital Affiliated to Kunming Medical University, Kunming, Yunnan, China; ^6^ School of Public Health, Kunming Medical University, Kunming, Yunnan, China

**Keywords:** BIRC5, cancer prognosis, tumor immune microenvironment, diagnostic biomarker, lung adenocarcinoma

## Abstract

**Introduction:**

BIRC5 (Survivin) is a crucial anti-apoptotic protein overexpressed in various cancers, promoting tumor growth and treatment resistance. This study investigates its expression across 33 cancer types and explores its diagnostic, prognostic, and immune-related significance.

**Methods:**

We analyzed RNA-seq data from TCGA and protein expression data from the Human Protein Atlas. Expression levels were compared between tumor and normal tissues. Correlations with molecular and immune subtypes were explored using TISIDB. Prognostic significance was evaluated through survival analysis, Cox regression, and ROC curve analysis. The PPI network was constructed using STRING.

**Results:**

BIRC5 was significantly overexpressed in tumor tissues across 33 cancer types, with higher expression levels observed in tumors compared to normal tissues. The protein expression analysis revealed a similar trend. BIRC5 expression was significantly correlated with various molecular and immune subtypes in multiple cancer types. Survival analysis indicated that high BIRC5 expression was associated with poor prognosis across multiple cancers, including lung adenocarcinoma (LUAD) and kidney renal clear cell carcinoma (KIRC). ROC analysis showed that BIRC5 exhibited strong diagnostic potential, with high AUC values (>0.9) in several cancers. The PPI network analysis identified key interacting proteins involved in the cell cycle and tumor progression, further supporting BIRC5's role in cancer biology. Functional experiments in lung adenocarcinoma (LUAD) revealed that BIRC5 upregulation enhances cell proliferation, migration, and invasion, while its knockdown suppresses these activities.

**Discussion:**

BIRC5 is a promising diagnostic and prognostic biomarker in multiple cancers. Its association with immune subtypes suggests a potential role in the tumor immune microenvironment. These findings support BIRC5 as a therapeutic target for cancer treatment.

## 1 Introduction

Cancer remains one of the leading causes of mortality worldwide, with its complex biological processes posing significant challenges for diagnosis, treatment, and prognosis (Ambeskovic et al., 2024). The heterogeneity of malignant tumors is evident in various aspects, including diverse gene expression profiles and the complexity of the tumor microenvironment (Contini et al., 2024). This heterogeneity not only limits the effectiveness of conventional treatments but also elevates the risk of recurrence and complicates therapeutic approaches (Yuan et al., 2024). In recent years, advancements in molecular biology and genomics have progressively elucidated the molecular mechanisms underlying cancer initiation and progression, fostering the development of novel molecular markers for personalized diagnosis and targeted therapy (Fujiwara et al., 2024; Gao T. et al., 2024).

BIRC5 (Baculoviral IAP Repeat Containing 5), also referred to as Survivin, is a key member of the inhibitor of apoptosis protein (IAP) family (Broodman et al., 2016). Survivin plays a crucial role in several fundamental biological processes, including cell cycle regulation, apoptosis inhibition, and cytokinesis, all of which are essential for tumor cell survival and proliferation (Chandrasekaran et al., 2022). Aberrant expression of BIRC5 is strongly associated with increased tumor cell proliferation, enhanced resistance to apoptosis, and treatment resistance (Bai et al., 2022). BIRC5 exerts its effects primarily by modulating the cell cycle and inhibiting apoptosis. It promotes cell cycle progression by interacting with cell cycle proteins and kinases (Zhang et al., 2024), while simultaneously preventing apoptosis by inhibiting key factors within the apoptotic pathway, such as caspase family proteins, thereby promoting tumor growth and survival (Gao B. et al., 2024). Moreover, Survivin is implicated in the regulation of several critical signaling pathways, including the p53 and Wnt/β-catenin pathways, highlighting its pivotal role in tumorigenesis and progression (Sanchon-Sanchez et al., 2023; Xia et al., 2015). However, the precise mechanisms by which BIRC5 influences various cancer types, particularly its expression across various molecular and immune subtypes and its clinical relevance, remain incompletely understood and warrant systematic investigation.

The aim of this study is to comprehensively analyze the expression patterns of BIRC5 across different cancer types using bioinformatics tools and databases. Specifically, we seek to investigate the relationship between BIRC5 expression and tumor subtypes, clinical prognosis, and molecular pathways. By utilizing publicly available datasets, such as TCGA and CCLE, we will assess the correlation between BIRC5 expression profiles and patient outcomes, as well as their potential implications for treatment responses. Ultimately, this research aims explore its potential as a diagnostic and therapeutic target.

## 2 Methods

### 2.1 Analysis of BIRC5 mRNA expression in tissues and cells

RNAseq data from 33 tumor types were downloaded from the TCGA database (https://portal.gdc.cancer.gov) and processed using the STAR pipeline. The data were extracted in TPM format, with matched paraneoplastic and carcinoma samples corresponding to numbered pairs. Analyses were performed using R software (version 4.2.1), with relevant packages including ggplot2 [3.3.6], stats [4.2.1], and car [3.1–0]. Data processing was conducted using the log2 (value + 1) transformation. Statistical analyses included the Wilcoxon rank-sum test and the Wilcoxon signed-rank test. Tumor cell line data were obtained from the Cancer Cell Line Encyclopedia (CCLE), and normal cell line data were retrieved from the BioGPS database (http://biogps.org).

### 2.2 Analysis of BIRC5 protein expression in tissues

To evaluate differences in BIRC5 protein expression, immunohistochemistry (IHC) images of BIRC5 expression in both normal and tumor tissues were downloaded from the Human Protein Atlas (HPA) (http://www.proteinatlas.org). This analysis included various cancer types, such as lymphoma, skin cancer, liver cancer, breast cancer, and lung cancer.

### 2.3 BIRC5 expression in molecular and immunological subtypes of tumors

The correlation between BIRC5 expression and various molecular and immune subtypes across different cancers was examined using the TISIDB database (http://cis.hku.hk/TISIDB/). Data on BIRC5 expression across different molecular and immune subtypes were downloaded from the “Gene” section of the database, and correlations between BIRC5 expression and specific cancer subtypes were subsequently analyzed.

### 2.4 Protein-protein interaction (PPI) network construction

To identify potential proteins interacting with BIRC5, the STRING database (https://string-db.org/) was utilized, and the top 20 proteins with the highest interaction probabilities were selected. The resulting protein-protein interaction network was visualized using Cytoscape software (version 3.9.0).

### 2.5 Gene ontology (GO) and Kyoto Encyclopedia of Genes and Genomes (KEGG) enrichment analyses

GO and KEGG enrichment analyses were conducted using the clusterProfiler package [4.4.4], following ID conversion of the input gene list via the org. Hs.e.g.,.db R package.

### 2.6 Analysis of diagnostic value

The diagnostic potential of BIRC5 in pan-cancer was assessed through receiver operating characteristic (ROC) curve analysis. RNAseq data from the pan-cancer STAR project were downloaded from the TCGA database, and data not corresponding to clinical information were excluded. The analysis was conducted using the pROC package [1.18.0], and the results were visualized using ggplot2 [3.3.6].

### 2.7 Survival prognosis analysis

Kaplan-Meier plots were generated to evaluate the relationship between BIRC5 expression and cancer prognosis, including overall survival (OS), progression-free interval (PFI), and disease-free survival (DSS). Proportional hazards hypothesis testing and survival regressions were conducted using the survival package [3.3.1], with visualization performed using the survminer package [0.4.9] and ggplot2 [3.3.6]. Cox regression analysis was applied, and a significance threshold of p < 0.05 was set.

### 2.8 Relationship between BIRC5 expression and clinical features in lung adenocarcinoma (LUAD)

RNAseq data from the TCGA-LUAD project were downloaded, processed in TPM format, and paired with corresponding clinical data. Cox regression analysis was performed using the survival package [3.3.1] to test the proportional hazards hypothesis and identify significant variables for inclusion in the multivariate Cox model.

### 2.9 Risk factor models for survival prediction

To predict patient survival, a nomogram was constructed, incorporating clinicopathological factors such as age, sex, disease type, stage, smoking status, alcohol consumption, BMI, and IRSS, using the rms package. ROC curves were employed to evaluate the predictive accuracy of the model. LASSO (least absolute shrinkage and selection operator) regression was performed to refine the model by reducing overfitting and improving interpretability. Additionally, one-way Cox regression identified 9 genes associated with lung cancer prognosis, which were incorporated into the final risk factor model.

### 2.10 Cell culture

Normal lung epithelial cells BEAS-2B and two lung cancer cell lines (A549, H1299) were cultivated in DMEM high-sucrose medium containing 10% FBS at 37°C and 5% CO_2_. Adherent cells were trypsinized when reaching 80%–90% confluence, counted, passaged, and replated. Suspended cells were directly resuspended, collected, centrifuged, and then the cell suspension was uniformly distributed before plating.

### 2.11 Western blot (WB)

We used RIPA buffer (1% PMSF) to lysed the cells, and BCA method was used to determine the concentration of proteins in cells or tissues. Proteins were separated by 7.5% SDS-PAGE, transferred to a PVDF membrane, and incubated with primary and secondary antibodies against the corresponding proteins. Band analysis was conducted post-exposure under developer.

### 2.12 Cellular *in vitro* experiments

Cells at 40%–60% fusion were transfected with lentivirus (Knockdown (KD) and overexpression (OE)) for 48 h. Cells at 40%–60% confluence were transfected with inhibitor BIRC5 for 48 h. Transfection efficiency was observed using fluorescence microscopy and WB. The cells (A549, A549-KD-BIRC5, A549-OE-BIRC5) were incubated in 96-well plates for 0, 1, 3, and 5 days respectively, and performed with Cell Counting Kit-8 (CCK8) assay. Cells (A549, A549-KD-BIRC5, A549-OE-BIRC5) were inoculated into Culture-Insert with scratches up to 500 μm wide. The length of scratches was observed and recorded under the microscope after culturing for 0, 24, 48, and 72 h. The area of scratched area was quantified using ImageJ software. The matrix gel was diluted at a ratio of 1:8, and 100 μL of the diluted matrix gel was added to the upper chamber of the Transwell plate, and incubated for 30 min at 37°C. Cell suspension of 200 μL (5 × 104 cells) is added to the upper chamber, and 500 μL of complete medium containing 20% FBS was added to the lower chamber. The plates were incubated for 48 h. The cells were gently wiped off the surface of the matrix gel and the transwell was fixed with 4% paraformaldehyde, and stained with 0.1% crystal violet. Six random fields were counted under the light microscope.

After trypsin digestion of cells in the logarithmic growth phase, resuspend the cells in complete medium (basal medium +10% fetal bovine serum) into cell suspension and inoculate each experimental group with 1,000 cells/well in 6-well plate culture. After 14 days of cloning culture, 1 mL of 4% paraformaldehyde was added to each well for 60 min, and the cells were washed twice with PBS. Add 1 mL of crystal violet staining solution to each well and stain the cells for 20 min. Wash the cells several times with PBS, dry them and take pictures under a light microscope.

## 3 Results

### 3.1 BIRC5 was differentially expressed in various tumors and normal tissues

**FIGURE 1:**
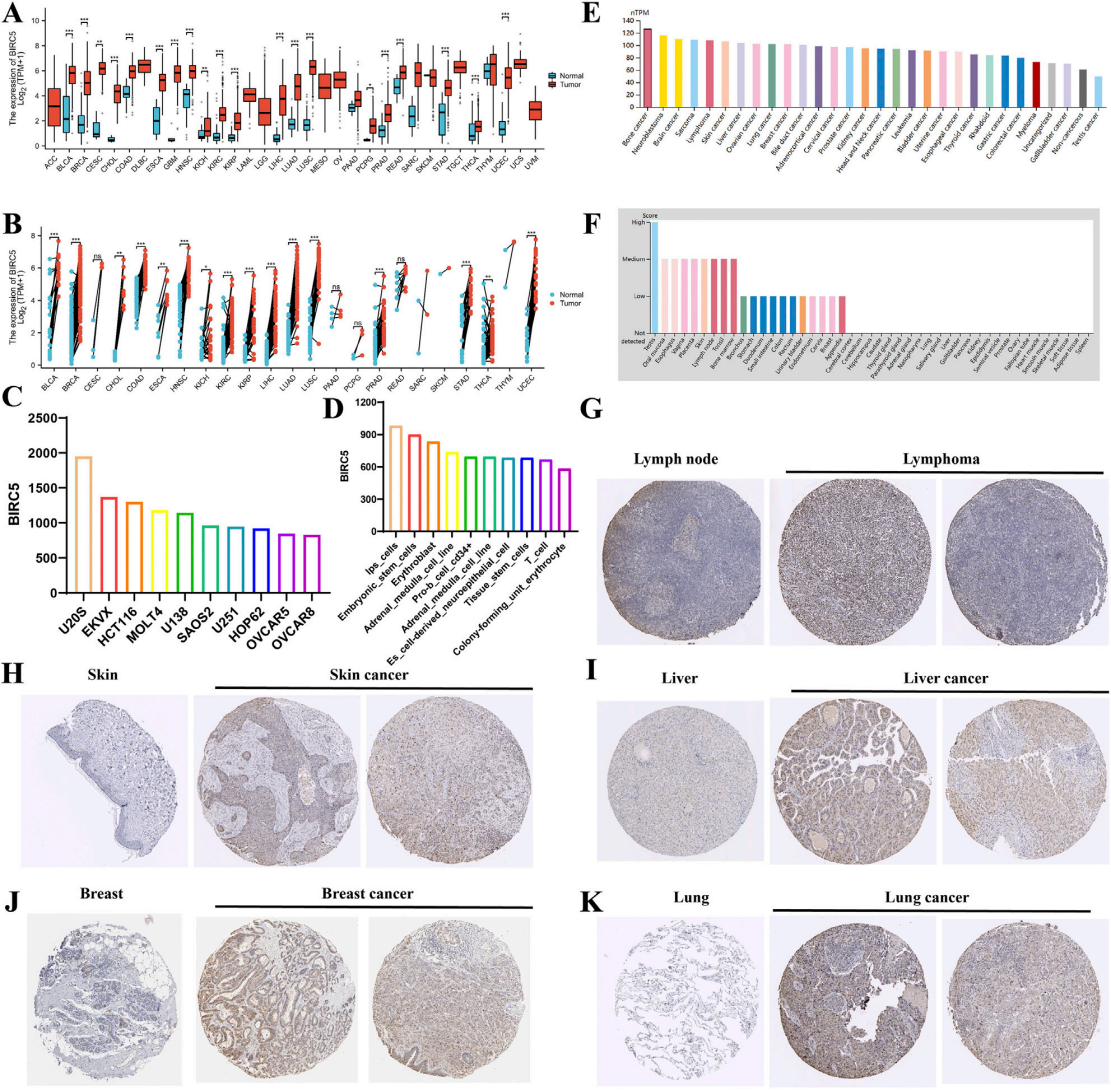
Differential analysis of BIRC5 expression in tumors and non-tumorous tissues. **(A)** BIRC5 mRNA expression in normal tissues and tumor cell lines (unpaired analysis). **(B)** BIRC5 mRNA expression in normal tissues and tumor cell lines (paired analysis). **(C)** BIRC5 mRNA expression in cancer cell lines. **(D)** BIRC5 mRNA expression in normal cell lines. **(E)** BIRC5 protein expression in tumor tissues from the HPA database. **(F)** BIRC5 protein expression in normal tissues from the HPA database. **(G)** BIRC5 expression in lymphoid tissues and lymphoid cancers. **(H)** BIRC5 expression in skin tissues and skin cancers. **(I)** BIRC5 expression in liver tissues and liver cancers. **(J)** BIRC5 expression in ovarian tissues and ovarian cancers. **(K)** BIRC5 expression in lung tissues and lung cancers (*p < 0.05, **p < 0.01, ***p < 0.001).

Comprehensive analysis of tumor databases revealed that the mRNA expression level of BIRC5 was significantly higher in tumors compared to corresponding normal tissues in all relevant unpaired samples, including BLCA, BRCA, CESC, CHOL, COAD, and 18 other tumor types (Figure 1A). Similar results were observed in paired samples, such as BLCA, BRCA, CHOL, and COAD across 16 tumor types (Figure 1B). Further analysis of BIRC5 expression in various cancer and normal cell lines through the BioGPS database indicated that BIRC5 was highly expressed in multiple cancer cell lines, whereas its expression was low in normal cell lines. Notably, BIRC5 expression was elevated in stem cells and immune cells (Supplementary Table S1). The top 10 cancer and normal cell lines with the highest BIRC5 mRNA expression levels are depicted in Figures 1C, D. To assess BIRC5 expression at the protein level, we analyzed immunohistochemistry (IHC) results from the HPA database and compared them with gene expression data. The mRNA expression data from Figures 1A, B did not include lymphoma and ovarian cancer, whereas the protein expression data from the HPA database, shown in Figures 1G–K, includes lymphoma (for example, in lymphoid tissues) and other cancers (such as liver, skin, and lung). These findings suggest that while the mRNA expression analysis did not include lymphoma and ovarian cancer, the protein expression data reveal significant upregulation of BIRC5 in these and other cancers. This highlights the importance of considering both mRNA and protein expression levels in understanding the role of BIRC5 in cancer progression. These findings suggest that the upregulated expression of BIRC5 in tumors may play a role in cancer initiation and progression.

### 3.2 Correlation between BIRC5 and tumor molecular and immune subtypes

**FIGURE 2:**
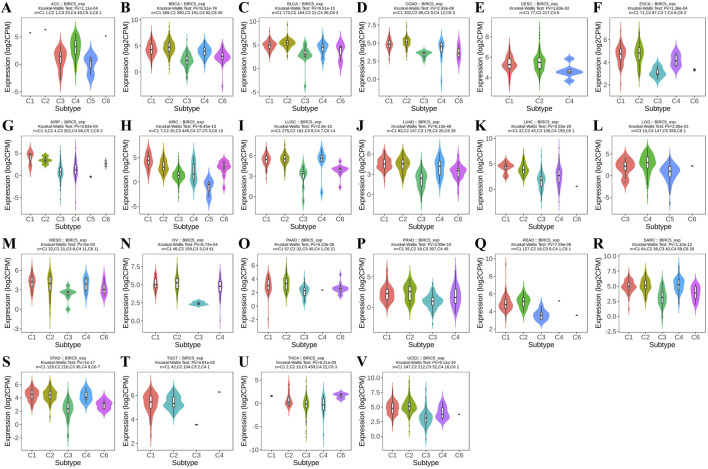
BIRC5 expression across different immunosubtypes in pan-cancer. **(A)** ACC, Adrenocortical Carcinoma; **(B)** BRCA, Breast Invasive Carcinoma; **(C)** BLCA, Bladder Urothelial Carcinoma; **(D)** COAD, Colon Adenocarcinoma; **(E)** CESC, Cervical Squamous Cell Carcinoma and Endocervical Adenocarcinoma; **(F)** ESCA, Esophageal Carcinoma; **(G)** KIRP, Kidney Renal Papillary Cell Carcinoma; **(H)** KIRC, Kidney Renal Clear Cell Carcinoma; **(I)** LUSC, Lung Squamous Cell Carcinoma; **(J)** LUAD, Lung Adenocarcinoma; **(K)** LIHC, Liver Hepatocellular Carcinoma; **(L)** LGG, Brain Lower Grade Glioma; **(M)** MESO, Mesothelioma; **(N)** OV, Ovarian Serous Cystadenocarcinoma; **(O)** PAAD, Pancreatic Adenocarcinoma; **(P)** PRAD, Prostate Adenocarcinoma; **(Q)** READ, Rectum Adenocarcinoma; **(R)** SARC, Sarcoma; **(S)** STAD, Stomach Adenocarcinoma; **(T)** TGCT, Testicular Germ Cell Tumors; **(U)** THCA, Thyroid Carcinoma; **(V)** UCEC, Uterine Corpus Endometrial Carcinoma (C1: wound healing, C2: IFN-gamma dominance, C3: inflammation, C4: lymphocyte depletion, C5: immune quiet, C6: TGF-b dominance), “Pv” refers to the p-value.

Using the TISIDB database, we observed that BIRC5 expression was significantly correlated with immune subtypes across most cancers (C1: wound healing, C2: IFN-gamma dominance, C3: inflammation, C4: lymphocyte depletion, C5: immune quiet, C6: TGF-b dominance), including ACC, BRCA, and BLCA, among 22 cancer types. Most tumors exhibited four to five immune subtypes, suggesting a strong link between BIRC5 and tumor immunity (Figure 2).

**FIGURE 3:**
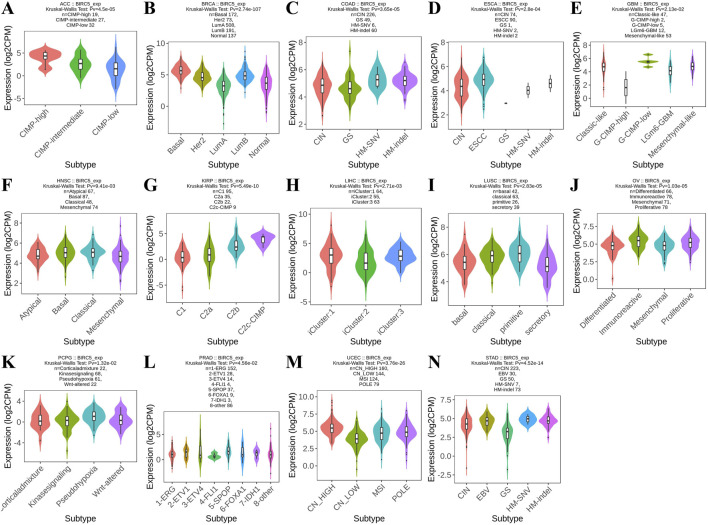
Analysis of BIRC5 molecular isoforms in pan-cancer. **(A)** ACC, Adrenocortical Carcinoma; **(B)** BRCA, Breast Invasive Carcinoma; **(C)** COAD, Colon Adenocarcinoma; **(D)** ESCA, Esophageal Carcinoma; **(E)** GBM, Glioblastoma Multiforme; **(F)** HNSC, Head and Neck Squamous Cell Carcinoma; **(G)** KIRP, Kidney Renal Papillary Cell Carcinoma; **(H)** LIHC, Liver Hepatocellular Carcinoma; **(I)** LUSC, Lung Squamous Cell Carcinoma; **(J)** OV, Ovarian Serous Cystadenocarcinoma; **(K)**. PCPG, Pheochromocytoma and Paraganglioma; **(L)** PRAD, Prostate Adenocarcinoma; **(M)** UCEC, Uterine Corpus Endometrial Carcinoma; **(N)** STAD, Stomach Adenocarcinoma. “Pv” refers to the p-value.

Furthermore, analysis revealed that BIRC5 expression varied significantly across molecular subtypes in 14 cancer types, including ACC, BRCA, COAD, ESCA, GBM, HNSC, KIRP, LIHC, LUSC, OV, PCPG, PRAD, UCEC, and STAD. Most of these cancers exhibited four to five distinct molecular subtypes, underscoring the strong association between BIRC5 expression and tumor classification (Figure 3). For clarity, the full names of the molecular subtype abbreviations are provided in Supplementary Table S2. These findings suggest that BIRC5 may serve as a valuable biomarker to inform personalized cancer treatment.

### 3.3 Prognostic value of BIRC5 in cancer

**FIGURE 4:**
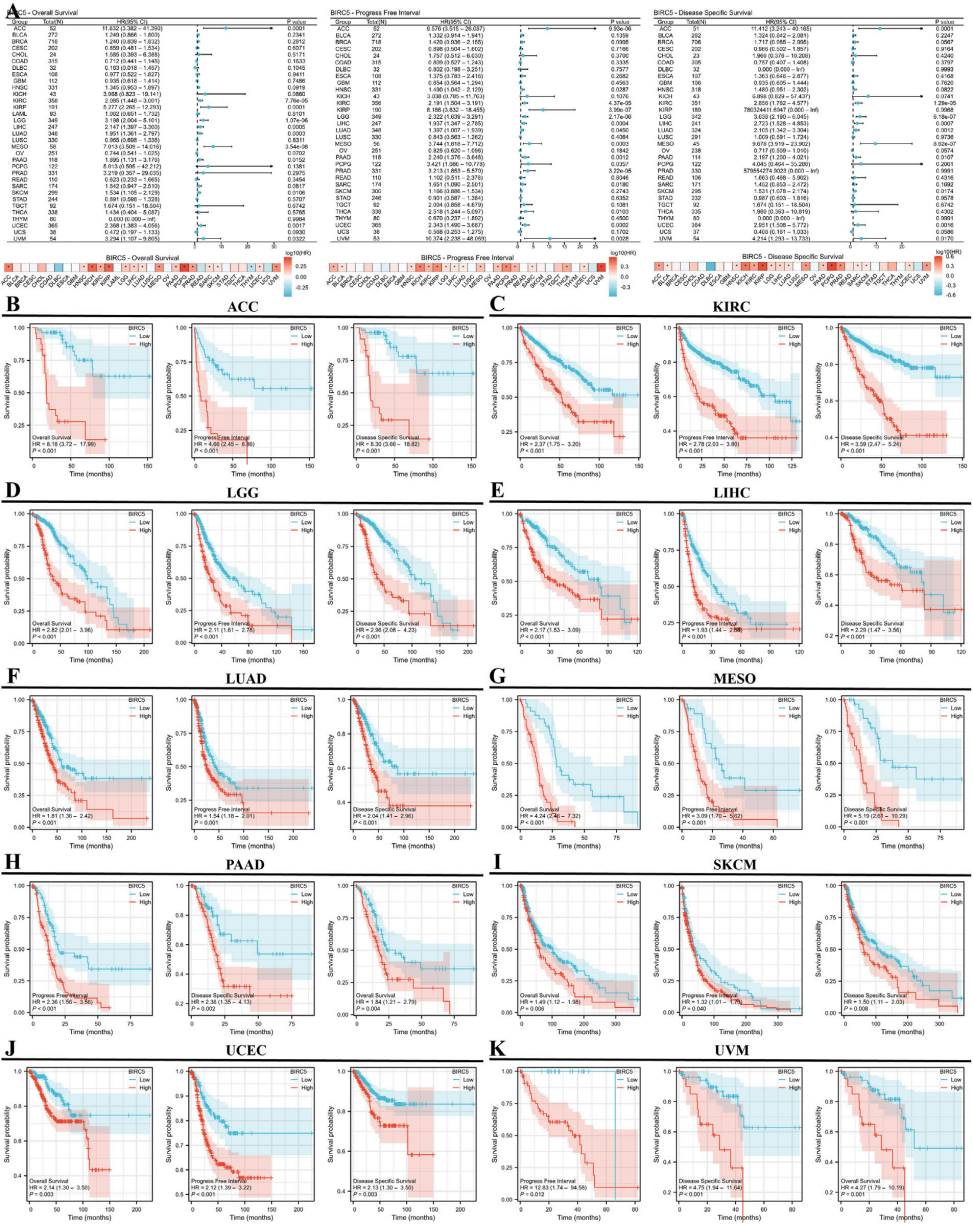
Prognostic and disease progression analysis of BIRC5 in pan-cancer. **(A)** Forest plot and heatmap summarizing BIRC5’s prognostic value (OS, PFI, DSS); **(B–I)** Kaplan-Meier (KM) curves illustrating BIRC5 expression and prognosis across various cancer types (ACC, KIRC, LGG, LIHC, LUAD, MESO, PAAD, SKCM, UCEC, UVM).

We conducted a survival analysis (OS, DSS, and PFI) across multiple cancer types based on BIRC5 expression. High and low BIRC5 expression groups were defined based on the [median expression level across samples]. Results were presented using forest and heat maps (Figure 4A). BIRC5 expression significantly impacted OS, DSS, and PFI in 10 cancers: ACC, KIRC, LGG, LIHC, LUAD, MESO, PAAD, SKCM, UCEC, and UVM (Figures 4B–K). For instance, in ACC, BIRC5 was associated with poor prognosis in PFI (HR = 4.66, 95% CI: 2.45–8.86, p < 0.001), OS (HR = 8.18, 95% CI: 3.72–17.99, p < 0.001), and DSS (HR = 8.30, 95% CI: 3.66–18.82, p < 0.001). Similarly, in KIRC, BIRC5 significantly affected PFI, OS, and DSS (HR = 2.78, 2.37, and 3.59, respectively, all p < 0.001). These findings suggest that BIRC5 is a risk factor that negatively influences prognosis across several cancers.

### 3.4 Prognostic and predictive relevance of BIRC5 in pan-cancer analysis

**FIGURE 5:**
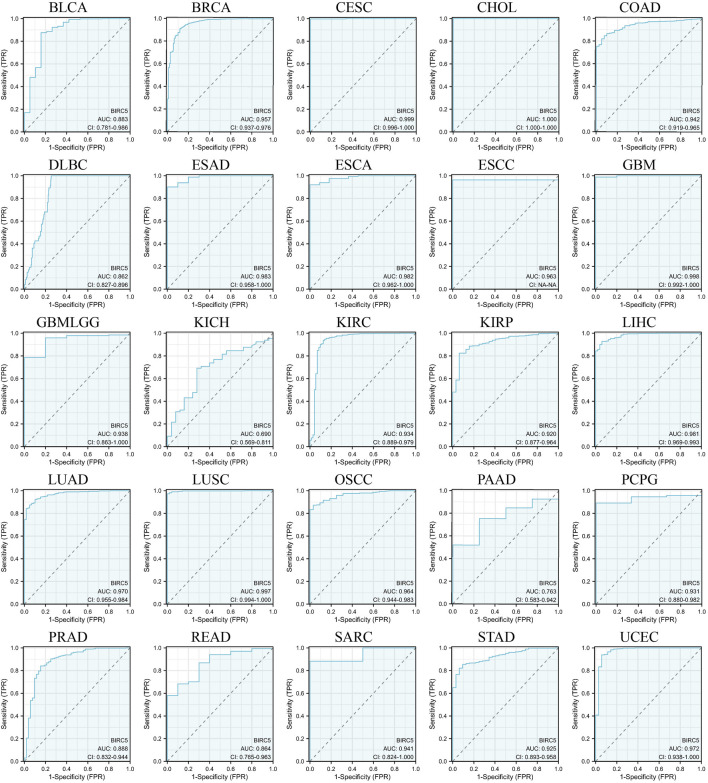
ROC curves evaluating the predictive and prognostic relevance of BIRC5 expression across multiple cancer types.

We evaluated the potential of BIRC5 to distinguish between cancerous and non-cancerous tissues across multiple cancer types using receiver operating characteristic (ROC) curves and area under the curve (AUC) values. Although ROC curves are commonly used to assess diagnostic capability, in this context, they provide insights into the broader relevance of BIRC5 in differentiating cancerous tissues and its potential role in prognosis across various cancer types. BIRC5 demonstrated a high AUC (>0.7) in 24 cancer types, including BLCA, BRCA, CESC, CHOL, COAD, DLBC, ESAD, ESCA, ESCC, GBM, GBMLGG, KIRC, KIRP, LIHC, LUAD, LUSC, OSCC, PAAD, PCPG, PRAD, READ, SARC, STAD, and UCEC (Figure 5). In 18 of these cancers, BIRC5 exhibited particularly strong predictive power (AUC > 0.9), including in BRCA, CESC, CHOL, COAD, ESAD, ESCA, GBM, LIHC, LUAD, and UCEC. These findings suggest that BIRC5 may serve as a valuable biomarker for distinguishing cancerous tissues and potentially predicting cancer progression, supporting its broader relevance in pan-cancer analysis.

### 3.5 Analysis of BIRC5 interacting proteins and their downstream functions

**FIGURE 6:**
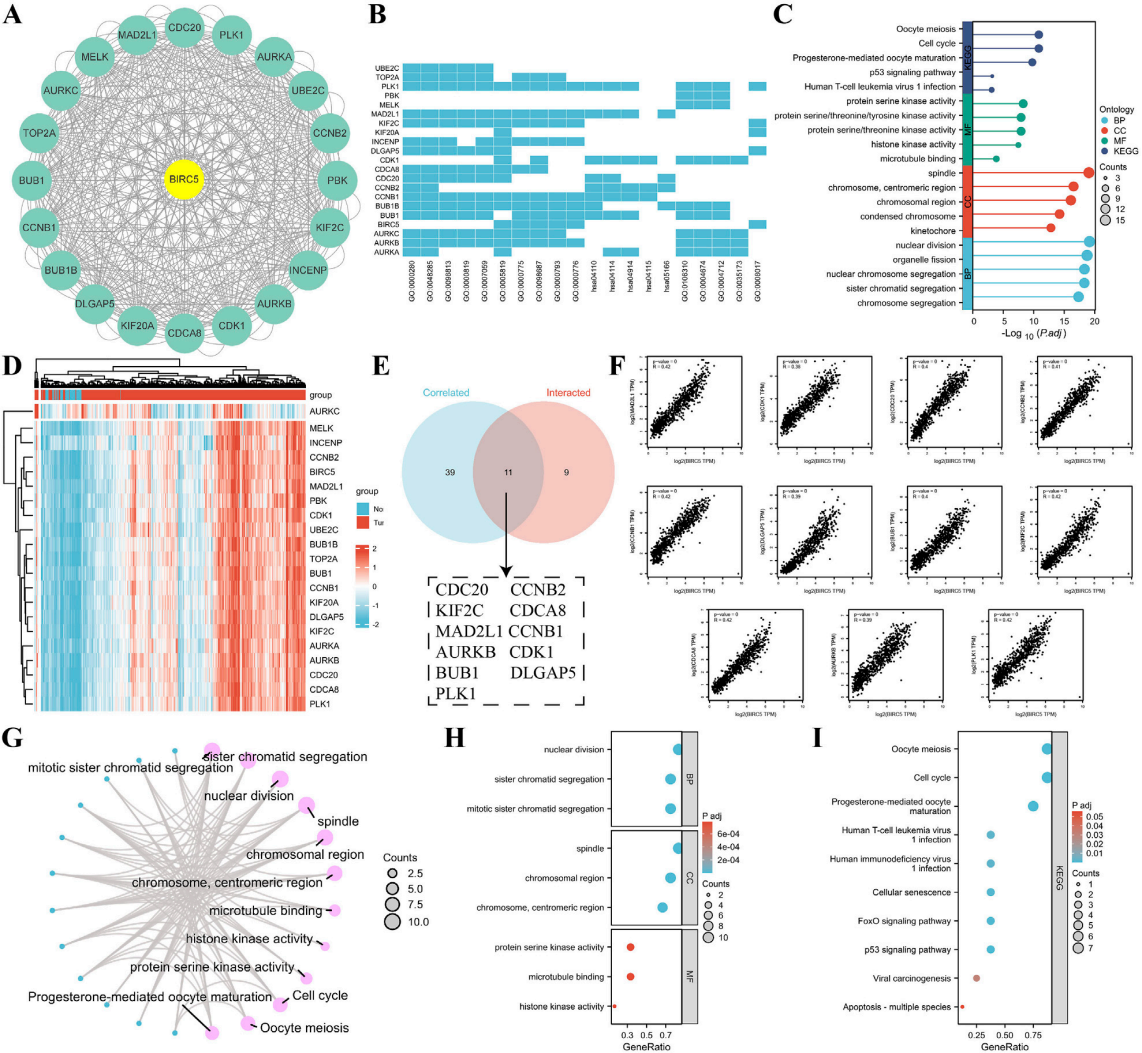
Correlation and enrichment analysis of BIRC5-related genes and proteins. **(A)** PPI network showing the top 20 proteins interacting with BIRC5; **(B–C)** Heatmap and lollipop plot of GO and KEGG analysis of BIRC5-related proteins; **(D)** Heatmap of the top 50 genes correlated with BIRC5 in lung cancer; **(E)** Venn diagram showing the overlap between the top 50 genes associated with BIRC5 in lung cancer datasets and STRING-derived proteins interacting with BIRC5; **(F)** Eleven genes highly correlated with BIRC5; **(G)** Network diagram of GO and KEGG pathways associated with BIRC5 and the 11 genes; **(H–I)** Bubble plot of GO and KEGG analysis of BIRC5 and its interacting proteins.

Using the STRING database and Cytoscape, we identified the top 20 proteins interacting with BIRC5 (Figure 6A). Gene Ontology (GO) enrichment analysis of these proteins revealed key biological processes, including nuclear division, organelle fission, and chromosome segregation. Cellular components involved the spindle and chromosomal regions, while molecular functions included protein serine kinase and histone kinase activity. KEGG pathway enrichment highlighted processes related to the cell cycle, oocyte meiosis, p53 signaling, and viral infections (Figures 6B, C).

To explore BIRC5’s specific role in LUAD, we identified the top 50 genes associated with BIRC5 in lung cancer datasets (Supplementary Table S3). These genes were cross-referenced with STRING-derived proteins, yielding 11 interacting proteins, including CDC20, KIF2C, and AURKB, all of which were significantly correlated with BIRC5 (Figures 6D–F). Functional enrichment analysis suggested that BIRC5 shares similar roles across various cancers, further supporting its involvement in key cancer pathways (Figures 6G–I).

### 3.6 Correlation between BIRC5 expression and immune cell infiltration in lung cancer

**FIGURE 7:**
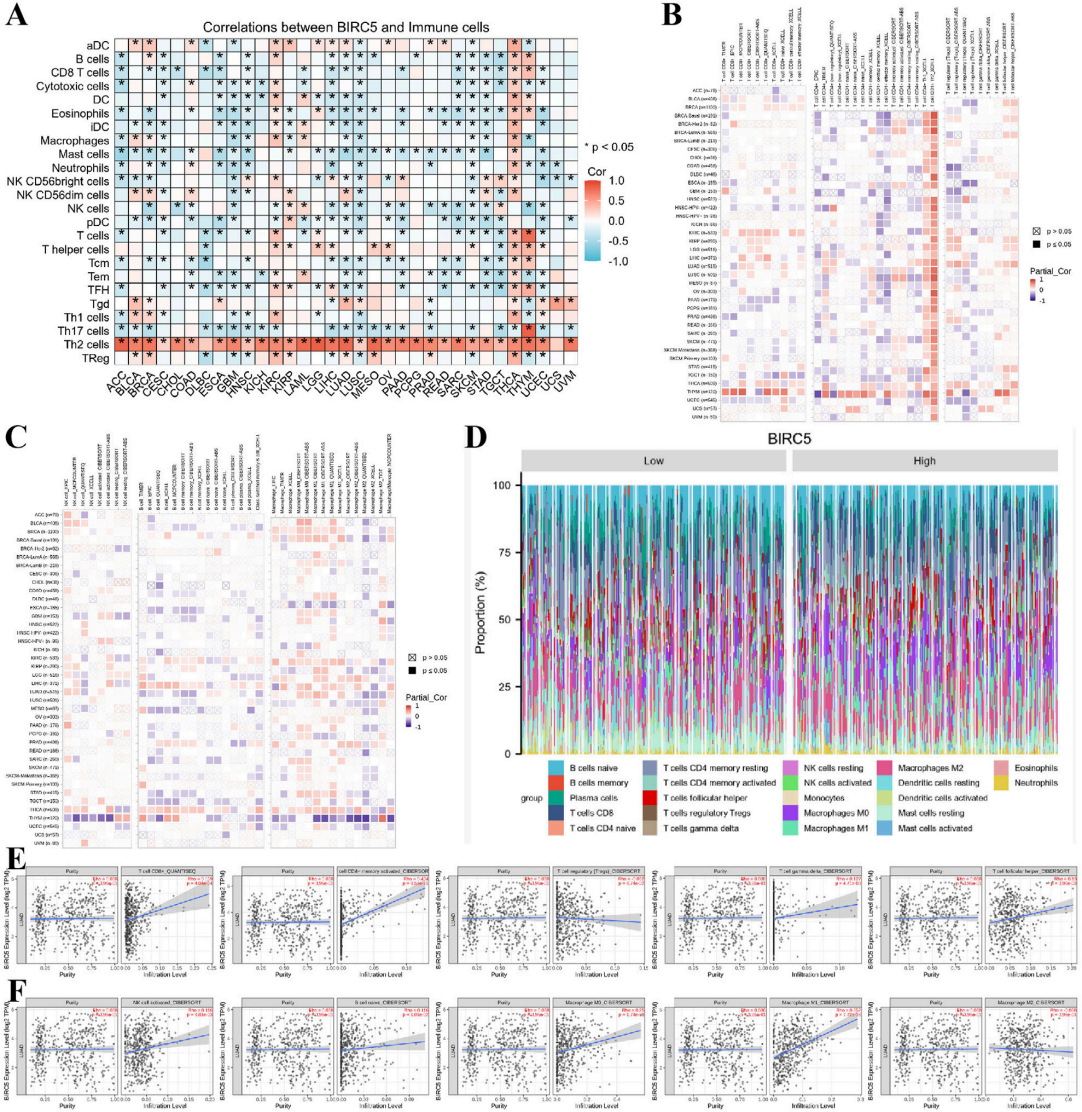
BIRC5 expression is closely associated with immune cell infiltration in cancer. **(A)** Heatmap of BIRC5 expression and immune cell infiltration across pan-cancer. **(B,C)** Heatmap of BIRC5 expression and the infiltration of T cells, B cells, macrophages, and NK cells across pan-cancer. **(D)** Stratified analysis of BIRC5 expression and immune cell infiltration in lung cancer. **(E,F)** Correlation analysis of T cells, B cells, macrophages, NK cells, and BIRC5 gene expression in lung cancer.

Previous functional studies have identified a strong association between BIRC5 and immune responses. To further explore this, we examined the relationship between BIRC5 expression levels and the infiltration of 26 immune-related cell types. The results from the heatmap analysis of BIRC5 expression and immune cell infiltration across various cancers indicated that BIRC5 exhibits immunosuppressive effects in many tumor types (Figure 7A). Subsequently, we performed a correlation analysis between BIRC5 expression and the infiltration levels of various immune cells, including T cells, B cells, macrophages, and NK cells. The analysis revealed a general trend where high BIRC5 expression was associated with a reduced infiltration of these immune cell subtypes in most cancers (Figures 7B, C). Focusing on lung cancer as a representative tumor type, we further analyzed the correlation between BIRC5 and immune cell infiltration. The stacked bar graph showed a positive correlation between BIRC5 expression and immune cell infiltration in lung cancer (Figure 7D). To validate these findings, we conducted an additional analysis using the TIMER 2.0 database, which confirmed that BIRC5 expression was significantly positively correlated with several immune cell types, including CD8^+^ T cells (Rho = 0.159), CD4^+^ T cells (Rho = 0.404), gamma delta T cells (Rho = 0.127), follicular helper T cells (Rho = 0.130), NK cells (Rho = 0.116), B cells (Rho = 0.116), macrophage M0 (Rho = 0.250), and macrophage M1 (Rho = 0.352). Only a few immune cell types, such as Tregs (Rho = −0.082) and macrophage M2 (Rho = −0.038), showed a negative correlation with BIRC5 expression (Figures 7E, F). These results are consistent with the pan-cancer analysis, reinforcing the idea that BIRC5 may serve as an effective intervention target in tumor immunotherapy.

### 3.7 BIRC5 and clinical subgroups of lung adenocarcinoma

**FIGURE 8:**
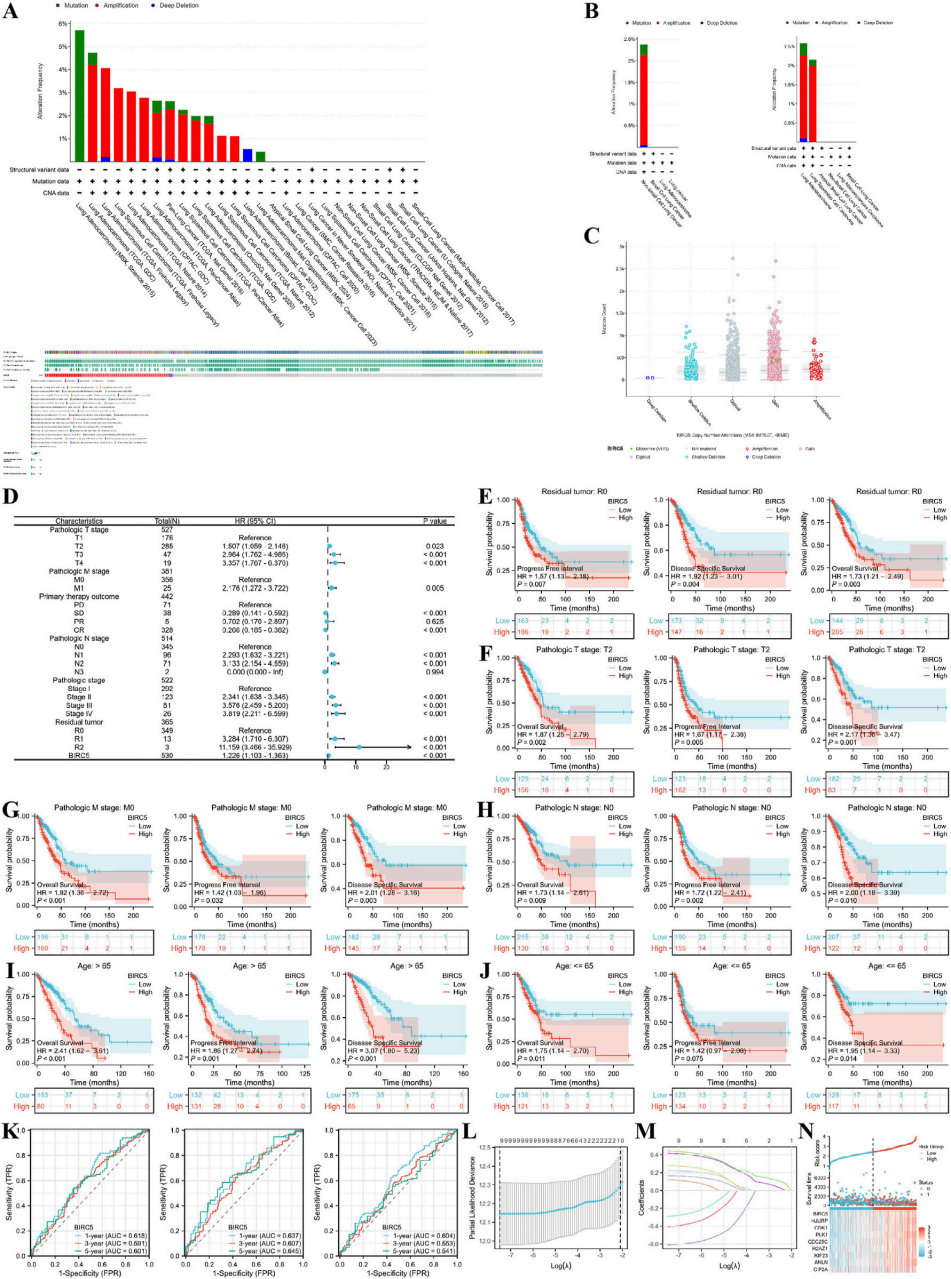
Genomic alterations of BIRC5 in lung cancer and their correlation with prognosis across clinical subgroups. **(A)** OncoPrint of BIRC5 alterations in cancer cohorts; **(B)** Detailed breakdown of BIRC5 gene alterations in lung cancer; **(C)** Major types of BIRC5 alterations; **(D)** Forest plot summarizing BIRC5’s prognostic value in different clinical subgroups; **(E–J)** Kaplan-Meier (KM) curves showing BIRC5 expression, prognosis, and disease progression in lung cancer, stratified by clinical factors (age, residual tumor status, etc.); **(K)** Time-dependent ROC curve predicting 1, 3, and 5-year survival; **(L,M)** LASSO model results with cross-validation for tuning parameter selection and coefficient profiles; **(N)** Risk score, survival status, and heatmap of eight genes in lung cancer patients.

We first explored the genomic alterations of BIRC5 in lung cancer using the cBioPortal website and found that BIRC5 genomic changes occurred in 2.4% of cases (Figure 8A). The types of alterations in BIRC5, including amplification, gain, and diploid states, resulted in changes in gene expression, with copy number variations (CNVs) being positively associated with gene expression in some sequencing results (Figures 8B, C). Next, we investigated the role of BIRC5 in patient prognosis and disease progression across various clinical features of lung cancer. High and low expression groups were determined based on the [median expression level of BIRC5 in the cohort]. The results are presented in the form of a forest plot (Figure 8D). BIRC5 was identified as a risk factor that promotes lung cancer progression, particularly in patients with Pathologic T2 stage, N0 stage, M0 stage, and residual tumor status (R0), as well as in patients of all age groups (Figures 8E–J). To determine whether BIRC5 can serve as an effective biomarker for lung cancer prognosis, we used receiver operating characteristic (ROC) curves to evaluate its predictive performance for overall survival (OS), disease-specific survival (DSS), and progression-free interval (PFI) in lung cancer patients at 1, 3, and 5 years. The results showed that all area under the curve (AUC) values were greater than 0.5, indicating that BIRC5 is a reliable prognostic predictor at multiple time points. Further analysis of genes co-expressed with BIRC5 in lung cancer revealed eight genes significantly associated with prognosis, as determined by univariate Cox regression analysis. LASSO regression analysis identified nine genes—BIRC5, HJURP, CDK1, PLK1, CDC25C, H2AZ1, KIF23, ANLN, and CIP2A—as significant risk factors impacting the prognosis of lung cancer patients (Figures 8L–N).

### 3.8 Co-expression analysis and functional enrichment of BIRC5 in lung adenocarcinomas

**FIGURE 9:**
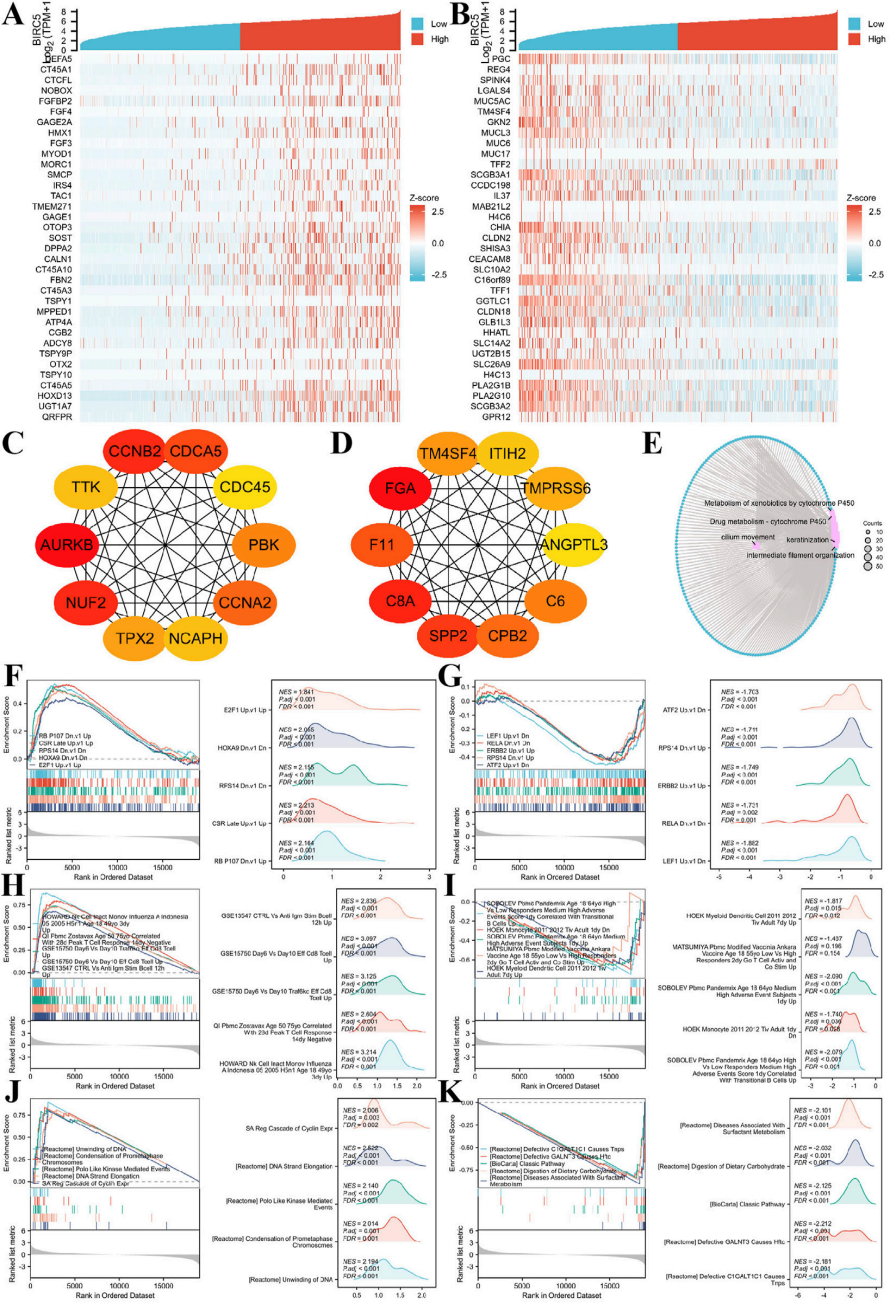
BIRC5 differential expression in lung cancer and functional enrichment analysis. **(A–B)** Heatmaps of the top 30 genes positively and negatively correlated with BIRC5 in lung cancer; **(C–D)**. HUB genes positively and negatively correlated with BIRC5; **(E)**. Network diagram of GO/KEGG analysis of BIRC5 co-expressed genes; **(F–G)** Visualization of GSEA results for BIRC5 co-expressed genes (oncogenic); **(H–I)** GSEA visualization of BIRC5 co-expressed genes in immunologic signatures; **(J–K)** GSEA visualization of BIRC5 co-expressed genes in gene ontology.

We investigated genes co-expressed with BIRC5 in LUAD, identifying those positively or negatively correlated with its expression. The top 30 positively and negatively correlated genes are displayed in the heatmap (Figures 9A, B). A total of 1,233 differentially expressed genes (DEGs) were identified, including 609 upregulated and 624 downregulated genes. Protein-protein interaction (PPI) networks were constructed for both upregulated and downregulated genes, revealing hub genes (Figures 9C, D).

Gene Ontology (GO) and Kyoto Encyclopedia of Genes and Genomes (KEGG) enrichment analysis of the DEGs showed that BIRC5 co-expressed differential genes in LUAD were primarily involved in processes such as cilium movement, intermediate filament organization, and keratinization. Cellular components (CC) enriched in these genes included the apical plasma membrane, apical part of the cell, and cornified envelope. Molecular functions (MF) were mainly associated with peptidase inhibitor activity, endopeptidase inhibitor activity, and structural components of the skin epidermis. KEGG pathway enrichment was predominantly linked to bile secretion, cytochrome P450-mediated drug metabolism, and metabolism of xenobiotics by cytochrome P450 (Figure 9E).

Finally, Gene Set Enrichment Analysis (GSEA) was performed to explore the correlation of BIRC5-related DEGs with oncogenic and immunologic signatures, as well as gene ontology. The top five positive/negative correlations are summarized in the figures. Oncogenic signatures were primarily related to “CSR_LATE_UP.V1_UP,” “RPS14_DN.V1_DN,” “NFE2L2. V2” and “HOXA9_DN.V1_DN” (Figures 9F, G). Immunologic signatures were associated with “HOWARD_NK_CELL_INACT_MONOV_INFLUENZA_A_INDONESIA_05_2005_H5N1_AGE_18_49YO_3DY_UP,” “GSE15750_DAY6_VS_DAY10_TRAF6KO_EFF_C8_TCELL_UP,” “GSE15750_DAY6_VS_DAY10_EFF_CD8_TCELL_UP,” “GSE18893_TCONV_VS_TREG_24H_TNF_STIM_UP,” and “GSE36476_CTRL_VS_TSST_ACT_72H_MEMORY_CD4_TCELL_YOUNG_DN” (Figures 9H,I). Gene ontology was primarily enriched in “REACTOME_FORMATION_OF_THE_CORNIFIED_ENVELOPE,” “REACTOME_KERATINIZATION,” “WP_RETINOBLASTOMA_GENE_IN_CANCER,” and “REACTOME_MITOTIC_SPINDLE_CHECKPOINT” (Figures 9J, K). These results suggest a strong association of BIRC5 with oncogenic and immunologic signatures, as well as key gene ontologies, supporting its potential as a tumor biomarker and therapeutic target.

### 3.9 BIRC5 is significantly overexpressed in lung adenocarcinomas and strongly associated with tumor progression

**FIGURE 10:**
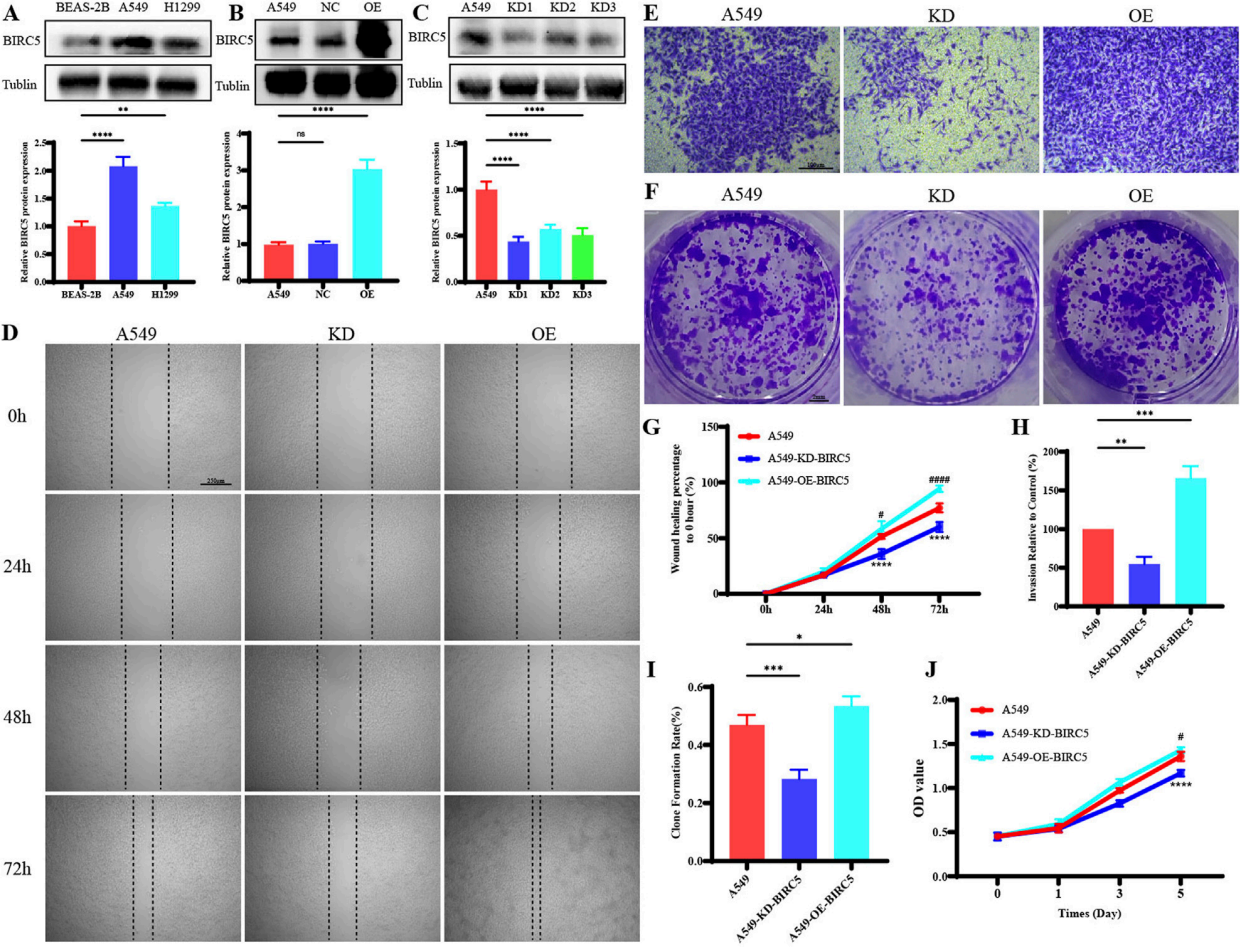
BIRC5 Affects Biological Functions in Lung Cancer **(A)** Protein expression of BIRC5 in normal lung epithelial cells and two lung cancer cell lines; **(B)** Detection of BIRC5 overexpression by Western blot analysis after transfection of BIRC5-specific or control oeRNA into A549 cells; **(C)** Detection of BIRC5 knockdown by Western blot analysis after transfection of BIRC5-specific into A549 cells; **(D)** Effect of BIRC5 on the migration ability of A549 cell; **(E)** Effect of IL20RB on the invasion ability of A549 cell; **(F)** Effect of BIRC5 on the proliferative capacity of A549 cells (plate clone); **(G)** Statistical analysis of cell migration ability results; **(H)** Statistical analysis of cell invasion ability results; **(I)** Statistical analysis of proliferative capacity results (plate clone); **(J)** Effect of BIRC5 on the proliferative capacity of A549 cells (CCK8).

In lung adenocarcinoma (LUAD), BIRC5 protein expression was significantly higher in tumor cell lines compared to normal lung epithelial cells (Figure 10A). Gene overexpression and knockdown assays in the A549 cell line demonstrated that inhibition of BIRC5 expression markedly reduced cell proliferation, migration, and invasion (Figures 10B–J), while its overexpression enhanced these tumorigenic properties. These findings indicate that BIRC5 is critically involved in the progression of LUAD and could serve as a potential therapeutic target.

## 4 Discuss

This study provides a comprehensive analysis of the expression patterns and clinical relevance of BIRC5 across a wide range of cancers. The findings indicate that BIRC5 mRNA levels are generally elevated in 33 tumor types compared to normal tissues, with significant protein overexpression in most cancerous tissues. Notably, in lung adenocarcinoma (LUAD), BIRC5 overexpression is strongly associated with poor prognosis across multiple clinical features. The ROC curve analysis demonstrates BIRC5’s robust diagnostic performance, with AUC values exceeding 0.9 in several cancer types, highlighting its potential as a cancer biomarker. Additionally, survival analysis reveals that high BIRC5 expression correlates with poorer outcomes in terms of overall survival (OS), progression-free survival (PFI), and disease-specific survival (DSS) across multiple cancers. Moreover, BIRC5 shows significant associations with both molecular and immune subtypes, with potential downstream mechanisms identified through protein-protein interaction (PPI) network analysis.

Compared with previous studies, this study not only verified the overexpression of BIRC5 in various cancers but also expanded the understanding of its clinical significance. Early studies mainly focused on the role of BIRC5 in individual cancer types, particularly in breast cancer (BRCA), gastric cancer (STAD), and liver cancer (LIHC), where BIRC5 overexpression was generally found to be closely associated with poor prognosis (Martínez-Sifuentes et al., 2022; Guo and Guo, 2024; Yang et al., 2023). For example, Ye et al. (2022) identified through bioinformatics analysis that BIRC5 overexpression in breast cancer was associated with tumor progression, drug resistance, and poor clinical prognosis (Ye et al., 2022). In addition, Fäldt Beding et al. (2022) observed high expression of BIRC5 in multiple cancer types in their pan-cancer study and suggested its potential as a prognostic biomarker, particularly showing strong clinical significance in gastric and liver cancers (Fäldt Beding et al., 2022). Xu et al. (2021) further explored the relationship between BIRC5 and the tumor immune microenvironment, finding that BIRC5 might play a crucial role in immune evasion by regulating immune cell infiltration, especially inhibiting T cell responses (Xu et al., 2021). The innovation of this study lies in its pan-cancer analysis, which not only confirmed the overexpression of BIRC5 across multiple cancers but also explored its potential role in tumor immune evasion. Our findings show a significant correlation between BIRC5 expression and various immune subtypes (e.g., C1: wound healing type, C2: IFN-γ dominant type, C3: inflammatory type), suggesting that BIRC5 may play a role in regulating immune cell infiltration and immune escape mechanisms. These results support the conclusions of previous studies by Ye et al., Fäldt-Beding et al., and Xu et al., which have linked BIRC5 to immune-related pathways and tumor immune microenvironment regulation. Notably, our pan-cancer analysis further strengthens the potential of BIRC5 as an immunotherapy target, particularly in cancers with significant immune evasion mechanisms.

The construction of the BIRC5 PPI network using the STRING database, along with GO and KEGG enrichment analyses, provided further insights into the biological processes and pathways involving BIRC5. The findings reveal that BIRC5 is actively involved in key processes such as cell cycle regulation, nuclear division, and chromosome separation, all of which contribute to cancer progression. Moreover, interactions between BIRC5 and other cancer-related proteins, including cell cycle regulators, apoptosis inhibitors, and DNA repair enzymes, underscore its significance in tumor biology (Long et al., 2024; Wijaya et al., 2024; Woo et al., 2022). GO analysis shows BIRC5’s involvement in regulating the cell cycle, cell division, and chromosome separation, consistent with its role in promoting cancer cell proliferation by inhibiting apoptosis. KEGG pathway analysis highlights that BIRC5 may contribute to cancer through its impact on the PI3K/Akt, MAPK, and NF-kB signaling pathways, all of which are crucial for tumor growth, survival, and metastasis. These pathways and protein interactions provide a clearer picture of the mechanisms by which BIRC5 promotes cancer progression.

Considering BIRC5’s high expression levels and poor prognostic implications, it holds promise as a marker for cancer diagnosis and prognosis. However, our study found that BIRC5’s prognostic significance may vary across cancer stages, with its impact being more pronounced in early-stage cancers, such as early-stage lung cancer, but less significant in later TNM stages. Consequently, BIRC5 could be integrated into cancer screening and prognosis assessment to enable more individualized and precise treatments. For example, detecting BIRC5 levels in cancers like breast, lung, and stomach could help predict patient outcomes and inform tailored therapeutic strategies. Future cancer treatments might involve targeted inhibition of BIRC5 to prevent tumor cell proliferation and improve patient outcomes. This could be achieved through small molecule inhibitors or antibodies that disrupt BIRC5’s interactions with other proteins, potentially improving the effectiveness of chemotherapy or immunotherapy regimens. This study also highlights BIRC5’s significant overexpression in lung adenocarcinoma (LUAD) and its strong association with tumor progression. These results provide direct evidence that BIRC5 plays a pivotal role in promoting tumor aggressiveness in LUAD. The inhibition of BIRC5 could therefore serve as a potential therapeutic strategy to limit tumor progression and improve patient outcomes in lung adenocarcinoma. This mechanistic insight further reinforces BIRC5’s potential as a prognostic marker and therapeutic target, particularly in cancers where its expression is closely linked to poor prognosis and aggressive tumor behavior. The clinical significance of LUAD, as the most prevalent subtype of non-small cell lung cancer (NSCLC), further highlights the rationale for this focus. Insights gained from studying LUAD may carry substantial clinical implications, particularly given the high mortality rate associated with this subtype. Expanding this analysis to include LUSC would provide a more comprehensive view of BIRC5’s role across lung cancer subtypes, serving as a logical continuation of this research.

While this study confirms BIRC5’s critical role in cancer progression and highlights its diagnostic and prognostic potential, further research is necessary to validate these findings and uncover the precise mechanisms by which BIRC5 operates. Future work should focus on the role of BIRC5 in immune escape mechanisms, assess its viability as a target for cancer therapies, and evaluate treatment strategies in clinical trials. Such research could eventually translate these findings into practical therapies, improving outcomes and survival for cancer patients.

## 5 Conclusions

This study highlights the significant overexpression of BIRC5 in various cancers, with particularly strong associations observed in lung adenocarcinoma, where BIRC5 is closely linked with tumor progression and poor prognosis. The correlation between BIRC5 expression and key molecular and immune subtypes, along with its interaction with critical proteins involved in cancer-related pathways, underscores its role in tumorigenesis. Moreover, *in vitro* functional experiments demonstrated that BIRC5 enhances the proliferative, migratory, and invasive capacities of lung cancer cells, supporting its potential as a therapeutic target. These findings demonstrate that BIRC5 is not only a robust biomarker for cancer diagnosis but also a promising target for the development of novel cancer therapies, particularly in lung adenocarcinoma.

## Data Availability

The original contributions presented in the study are included in the article/Supplementary Material, further inquiries can be directed to the corresponding authors.
